# Timing of cognitive decline in CLN3 disease

**DOI:** 10.1007/s10545-018-0143-x

**Published:** 2018-02-01

**Authors:** Willemijn F. E. Kuper, Claudia van Alfen, Roeliene H. Rigterink, Sabine A. Fuchs, Maria M. van Genderen, Peter M. van Hasselt

**Affiliations:** 1 Department of Metabolic Diseases, Wilhelmina Children’s Hospital, University Medical Center Utrecht, P.O. Box 85090, 3508 AB Utrecht, the Netherlands; 2 Bartiméus Institute for the visually impaired, Zeist and Doorn, the Netherlands

**Keywords:** CLN3 disease, Neuronal ceroid lipofuscinosis (NCL), Childhood neurodegeneration, Natural history

## Abstract

**Background:**

CLN3 disease is a major cause of childhood neurodegeneration. Onset of visual failure around 6 years of age is thought to precede cognitive deterioration by a few years, but casuistic reports question this paradigm. The aim of our study is to delineate timing of cognitive decline in CLN3 disease.

**Methods:**

Early neurocognitive functioning in CLN3 disease was analyzed using age at onset of visual and cognitive decline and IQ scores from literature-derived patient descriptions, supplemented with IQ scores and school history from a retrospective referral center cohort. We analyzed protracted and classical CLN3 separately and added a control group of patients diagnosed with juvenile onset macular degeneration (early onset Stargardt disease) to control for possible effects of rapid vision loss on neurocognitive functioning.

**Results:**

Onset of cognitive decline at a mean age of 6.8 years (range 2–13 years, *n* = 19) paralleled onset of visual deterioration at a mean age of 6.4 years (range 4–9 years, *n* = 81) as supported by an early decline in IQ scores in classical CLN3 disease. Onset and course of vision loss was similar in patients with protracted CLN3. The decreased IQ levels at diagnosis (mean 68.4, range 57–79, *n* = 9) in the referral cohort were consistently associated with an aberrant early school history contrasting normal school history and cognition in Stargardt disease patients.

**Conclusions:**

Cognitive dysfunction is universally present around diagnosis in classical CLN3 disease.

**Electronic supplementary material:**

The online version of this article (10.1007/s10545-018-0143-x) contains supplementary material, which is available to authorized users.

## Introduction

CLN3 disease (OMIM #204200), also known as juvenile neuronal ceroid lipofuscinosis or Batten disease, is the most frequent of the neuronal ceroid lipofuscinoses (NCLs). These genetically and clinically heterogeneous degenerative disorders of the brain and, in most forms, of the retina are unified by the intracellular accumulation of auto fluorescent storage material in most tissues. Collectively, they comprise a major cause of childhood neurodegeneration (Haltia 2003; Kousi et al. 2012).

Patients with CLN3 disease present with rapid visual decline around 6 years of age. It is generally believed that cognitive deterioration does not occur until several years after presentation (Kousi et al. 2012). Indeed, cognitive decline may not be apparent until well into adulthood in the protracted form of CLN3 disease displayed by a minority of patients (Lauronen et al. 1999; Munroe et al. 1997). Some casuistic reports, however, have suggested the presence of neuropsychological problems around, or even before, the onset of visual deterioration in the common classical phenotype (Kristensen and Lou 1983; Lamminranta et al. 2001; Spalton et al. 1980). It is presently unclear whether these problems should be attributed to the rapid vision loss or reflect early neurodegeneration.

To delineate timing of neurocognitive decline in CLN3 disease, we performed a systematic literature search and included the retrieved cases in a meta-analysis of case reports. Subsequently, we corroborated the data from the literature search with a cohort of patients diagnosed with CLN3 disease from the Dutch national NCL referral center and — to delineate the influence of rapid vision loss — we compared these to a cohort of patients with isolated retinal degeneration due to Stargardt disease.

## Methods

### Literature search

We searched PubMed and Embase for individual patients published until June 2017 with the search terms “CLN3 disease”, “juvenile neuronal ceroid lipofuscinosis”, “JNCL”, “juvenile NCL”, “batten disease”, “batten’s disease” “CLN3”, “NCL3”, “spielmeyer vogt sjogren”, “batten spielmeyer vogt”, “spielmeyer vogt”, “spielmeyer sjogren” and “juvenile amaurotic idiocy”. We manually checked the reference lists of included articles to identify additional suitable studies.

Patients were considered for inclusion if diagnosis of CLN3 disease was confirmed. Both genetic analysis confirming biallelic variants in *CLN3* and blood smear analysis revealing lymphocyte vacuolization, which is known to discriminate CLN3 disease from other NCL types, were considered sufficient proof (Anderson et al. 2013). Secondly, sufficient clinical data had to be retrievable (at least one out of: age at onset of visual failure, age at onset of cognitive decline, IQ test results). To avoid double inclusion of patients we matched patients if possible with their patient IDs from the UCL CLN3 mutation and patient databases (http://www.ucl.ac.uk/ncl/cln3.shtml) and we manually checked case reports on demographic and clinical characteristics. Patients displaying classical CLN3 disease were analyzed separately from a small subset displaying protracted CLN3 disease primarily based on genotype (Kousi et al. 2012; Munroe et al. 1997). If the genotype was unknown, patients were classified as classical unless the author explicitly classified the disease course as protracted.

For analysis, we extracted age at onset of visual failure, age at onset of cognitive decline, and IQ test results.

### Referral center cohort

To corroborate the findings from literature, we analyzed neurocognitive functioning around diagnosis in a referral center cohort of patients diagnosed from 1987 to 2016 with classical CLN3 disease. To control for possible effects of rapid early vision loss on neurocognitive functioning, we created a cohort of patients diagnosed with early onset Stargardt disease (OMIM #248200). Early onset Stargardt disease, presenting between 5 and 10 years, is associated with a similarly rapid loss of vision and was therefore considered a valid control group (Lambertus et al. 2015).

For data analysis, we extracted results from IQ tests performed around diagnosis and school history prior to and after diagnosis.

### Standard protocol approvals, registrations, and patient consents

A waiver of requirement for ethical review was granted by the Medical Ethical Committee at the University Medical Center Utrecht, the Netherlands.

### Data analysis

Kaplan-Meier curves were used to analyze age at onset of visual and cognitive deterioration in patients with either classical or protracted CLN3 disease. The mean was provided if the data were normally distributed; otherwise, the median was provided. Analysis of statistically significant differences in survival curves was performed using the log-rank test. Analysis of IQ scores was performed using Pearson r correlation for the overall decline and the paired t-test for the decline in IQ scores per patient. A *p*-value of <0.05 was considered significant. Prism software (version 7.02, Graphpad Software, San Diego, CA) was used for statistical analysis.

## Results

The literature search revealed 36 studies, describing 104 classical and 14 protracted CLN3 disease patients, that were eligible for inclusion (Suppl. Tables 1 and 2).

### Timing of cognitive decline

**Fig. 1:**
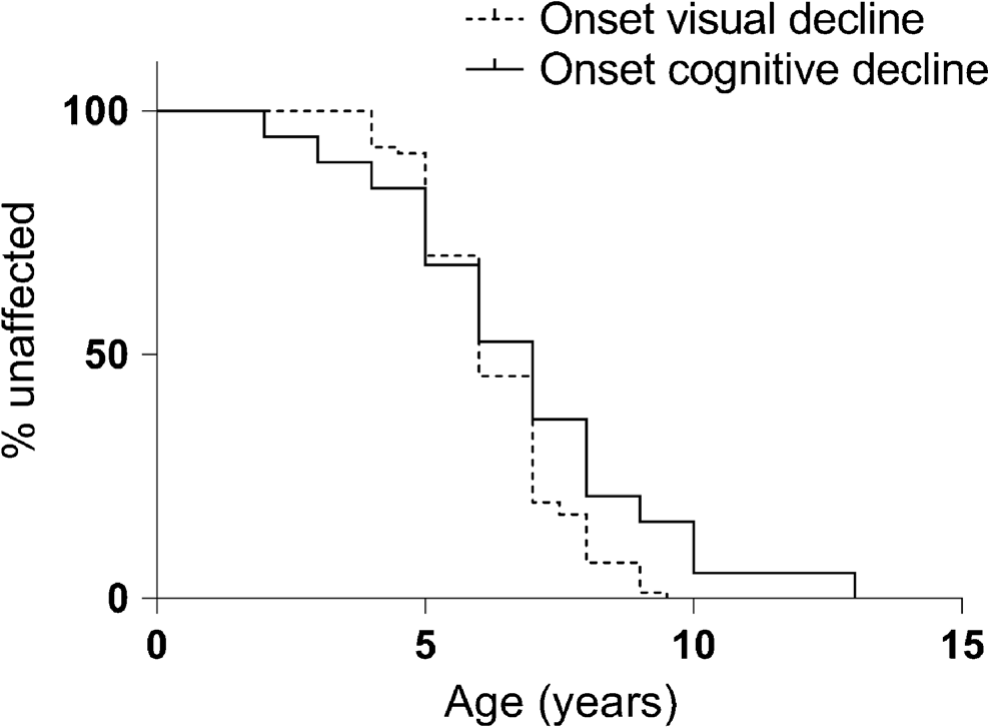
Onset of visual deterioration versus onset of cognitive decline in classical CLN3 disease. Kaplan-Meier survival curves of onset of cognitive decline (*n* = 19) versus onset of visual deterioration (*n* = 81) in classical CLN3 disease (*p* = 0.06)

**Fig. 2:**
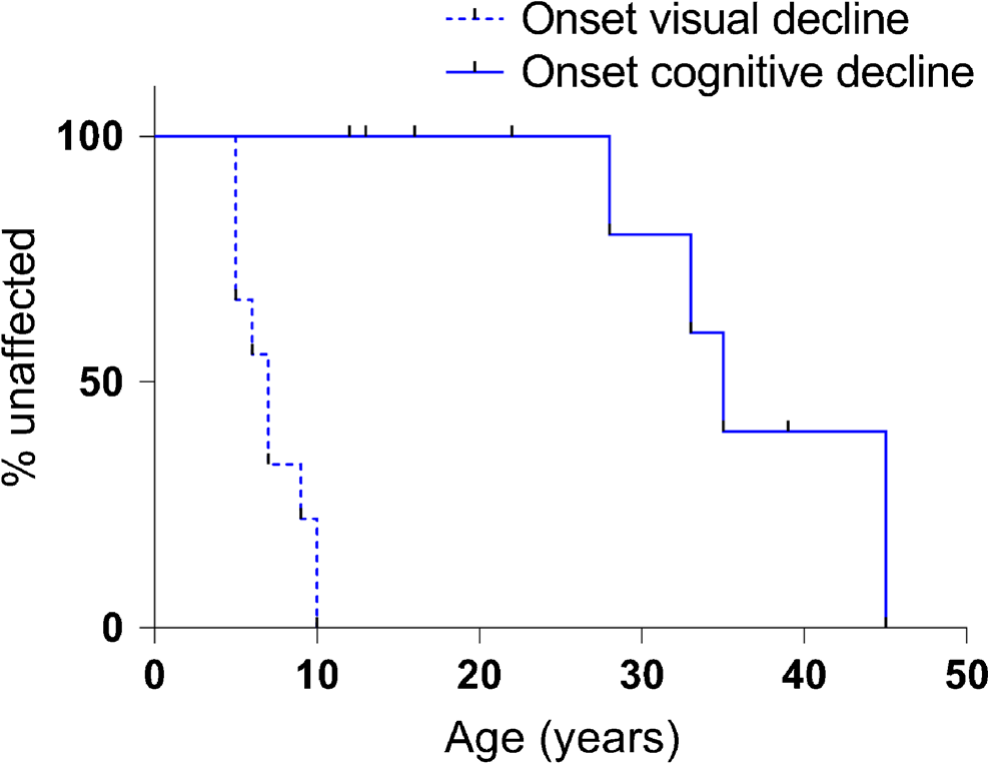
Onset of visual deterioration versus onset of cognitive decline in protracted CLN3 disease. Kaplan-Meier survival curves of onset of cognitive decline (*n* = 10 including censored cases) versus onset of visual deterioration (*n* = 9) in protracted CLN3 disease (*p* < 0.001)

Onset of cognitive decline at a mean age of 6.8 years (range 2–13 years, *n* = 19) paralleled onset of visual deterioration (mean age of 6.4 years, range 4–9 years, *n* = 81) in patients with classical CLN3 disease (Fig. 1). This finding remained when analysis was restricted to patients with genetically confirmed classical CLN3 disease (*n* = 73) (mean age at onset of visual deterioration (*n* = 50) and cognitive decline (*n* = 14): 6.2 years and 7.1 years, respectively). Onset of vision loss was only slightly later in protracted CLN3 disease (Fig. 2; mean age at onset 7.1 years, range 5–10 years, *n* = 9) similarly resulting in blindness within a few years after onset (data not shown) while cognition was unaffected around diagnosis and remained normal until adulthood.

**Fig. 3:**
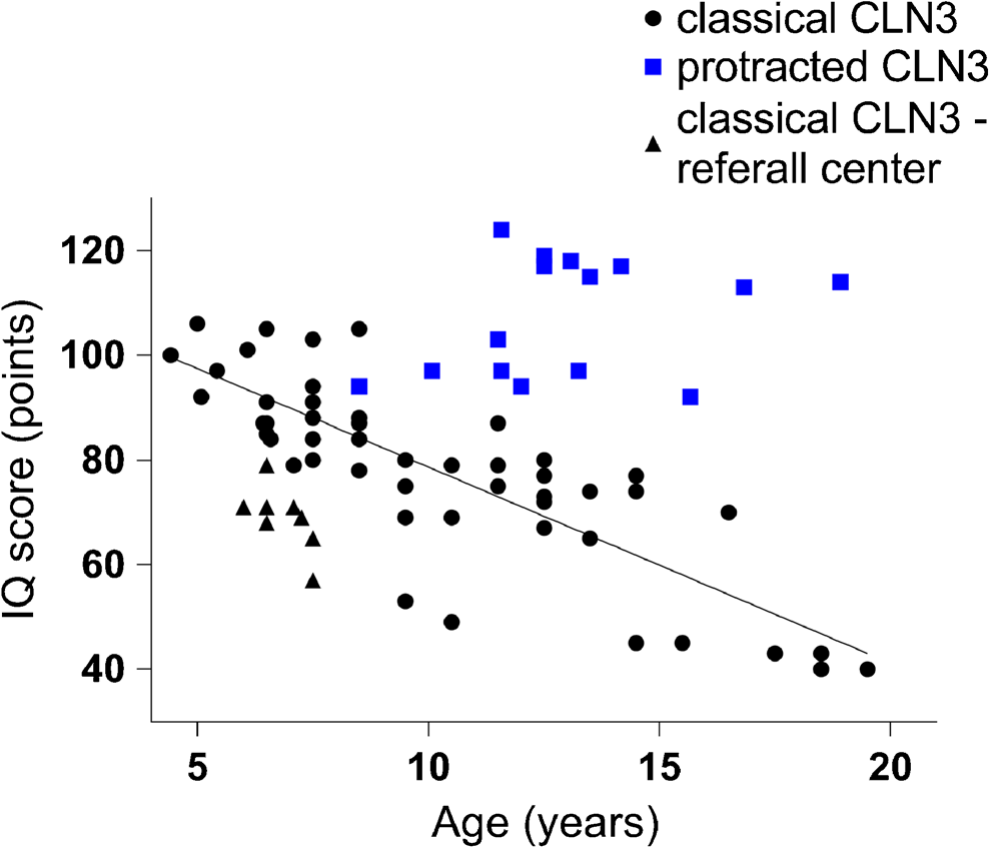
IQ scores in classical versus protracted CLN3 disease. IQ score decline in classical CLN3 disease versus IQ scores in protracted CLN3 disease. *r* = −0.8239 in classical CLN3 disease versus *r* = 0.3013 in protracted CLN3 disease

In patients with classical CLN3 disease, IQ scores declined from 91.4 (range 84–105, n = 7) at 6 years of age to 69.3 (range 53–80, *n* = 4) at 9 years of age (r-0.5596; p0.005), in line with the overall profound inverse correlation of IQ scores with age (*n* = 52 scores from 25 patients, r-0.8239, *p* < 0.001), contrasting the normal intelligence scores up to adulthood in protracted CLN3 disease (Fig. 3). This decline was also observed upon paired analysis of first and last IQ measurements in individual patients (from a mean IQ of 85.8 (49–106) to a mean IQ of 70.8 (40–88), p < 0.001).

These data indicated that cognitive decline in patients with classical CLN3 disease sets in appreciably earlier than expected. However, small sample size and the significant amount of missing data precluded a more definite conclusion. To corroborate these findings we therefore evaluated the timing of cognitive decline in a separate cohort of 21 patients diagnosed with classical CLN3 disease from the Dutch national NCL referral center and — to delineate the influence of rapid vision loss — we compared these to a cohort of 13 patients with isolated retinal degeneration due to early onset Stargardt disease.

**Fig. 4:**
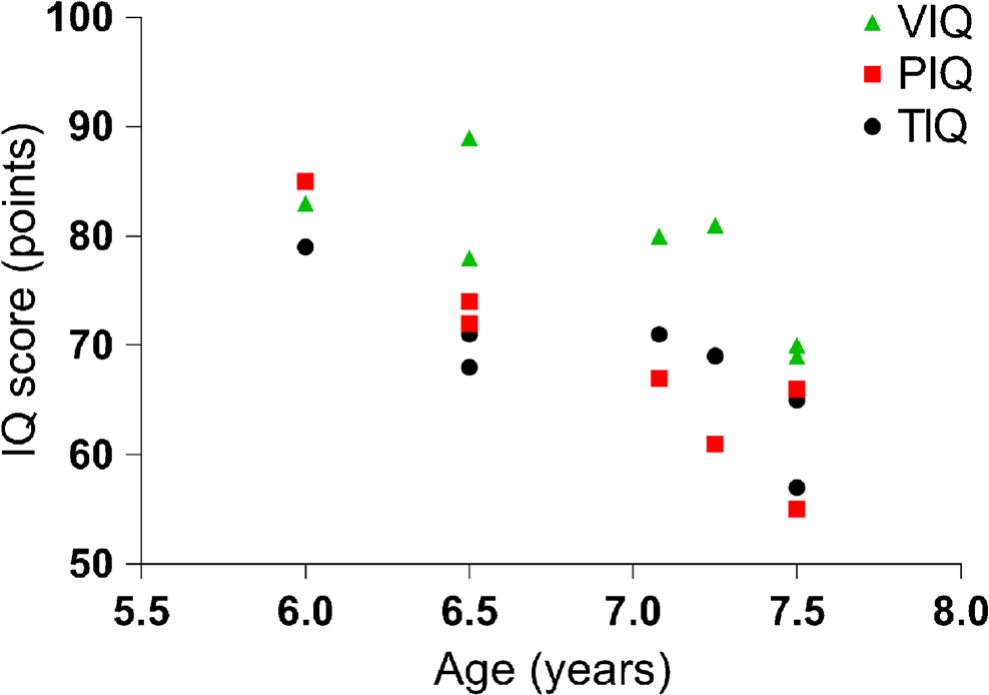
IQ scores split per IQ area in classical CLN3 disease around diagnosis. IQ score decline in classical CLN3 disease from the referral center cohort, split per IQ area. VIQ = verbal IQ; PIQ = performance IQ; TIQ = total IQ. Decline in VIQ: r-0.72 p0.0681. Decline in PIQ: r-0.92; p0.0031. Decline in TIQ: r-0.804; p0.0293

In this referral center cohort, IQ tests were performed around the time of diagnosis in 13 patients with classical CLN3 disease. In four patients the IQ tests could not be completed (due to aberrant behavior (1), attention deficit (1), unreliable, highly variable test results (1), and unspecified (1)). The IQ scores in the remaining nine patients were all well below average, in a range similar to, or even lower than, the range of patients derived from literature (mean 68.4, range 57–79, age 6–7 years) (Figs. 3 and 4). The decrease was more pronounced on performance than verbal subscales (Fig. 4). IQ tests around diagnosis in early onset Stargardt disease patients yielded normal results (*n* = 4: IQ scores of 95, 101, 96, and 100, respectively).

### Early cognitive functioning in daily life

In all classical CLN3 patients from whom information on school history was available (*n* = 18), additional educational support was provided before or at the age of 8 years explicitly noted due to learning difficulties. In contrast, all early onset Stargardt patients successfully completed regular level primary and secondary education solely requiring practical adjustments because of their visual impairment.

## Discussion

In this study, we evaluated timing and severity of cognitive decline in CLN3 disease in literature-derived patients supplemented with a representative referral center cohort. Our results show that in classical CLN3 disease cognitive deficits are consistently present around the time of diagnosis. In clinical practice, school problems may be commonly attributed to rapid decrease in vision. The unaffected (childhood) neurocognitive functioning in both protracted CLN3 disease and Stargardt disease patients with similarly early and severe visual deterioration delineates that low IQ test scores and poor school performance cannot be explained by severe vision loss alone.

The present study underlines that a meta-analysis of cases obtained through a systematic literature review allows further insight into the disease course of rare disease entities, if potential drawbacks are adequately addressed (Diekman et al. 2014). To address two major drawbacks in this study — missing data and publication bias — we validated the findings of the meta-analysis in a separate cohort. The IQ scores around diagnosis in our referral center cohort analysis demonstrate that the IQ scores found in literature do not represent patients with an extraordinary early age at onset of cognitive decline. If anything, they may underestimate — rather than overestimate — cognitive function around diagnosis in classical CLN3 disease. In both the literature-derived cases and the referral center cohort, a variety of IQ tests were used and it is uncertain whether or not these tests have been adjusted to the degree of visual impairment (Adams et al. 2007). This limitation, however, also applies to the other patients tested who, despite their visual impairment, all had normal IQ scores. Furthermore, the universal presence of early learning problems in classical CLN3 patients underscores that early intellectual impairment reflects actual functional impairment in daily life.

In summary, this study demonstrates that cognitive dysfunction is universally present around diagnosis in CLN3 disease, independent of the co-occurring vision loss. In a school aged child with rapid vision loss, learning difficulties should be perceived as an important cue for CLN3 disease.

## Electronic supplementary material

ESM 1 (DOCX 45 kb)
