# Supplementary material for: Timing of cognitive decline in CLN3 disease
Source: J Inherit Metab Dis. 2018 Feb 1;41(2):257–61. doi: 10.1007/s10545-018-0143-x (PMC5830481; doi:10.1007/s10545-018-0143-x)
Supplement: Supplementary file 1 — (DOCX 45 kb) [file 10545_2018_143_MOESM1_ESM.docx]

**Supplementary table 1: Summary of patient characteristics systematic review – classical CLN3**

| **Patient No** | **Mutation 1** | **Mutation 2** | **Vacuolated lymphocytes** | **Age onset visual decline (years)** | **Age onset cognitive decline**  **(years)** | **IQ scores** | **IQ test method used** |
| --- | --- | --- | --- | --- | --- | --- | --- |
| 1^1^ | 1.02kb del | 1.02 kb del | ? | 4 | ? |  |  |
| 2^1^ | 1.02 kb del | 1.02 kb del | ? | 8 | ? |  |  |
| 3^1^ | 1.02 kb del | 1.02 kb del | ? | 9 | ? |  |  |
| 4^1^ | 1.02 kb del | 1.02 kb del | ? | 7 | ? |  |  |
| 5^1^ | 1.02 kb del | 1.02 kb del | ? | 7 | ? |  |  |
| 6^1^ | 1.02 kb del | 1.02 kb del | ? | 4 | ? |  |  |
| 7^1^ | 1.02 kb del | 1.02 kb del | ? | 6 | ? |  |  |
| 8^1^ | 1.02 kb del | 1.02 kb del | ? | 4 | ? |  |  |
| 9^1^ | 1.02 kb del | 1.02 kb del | ? | 7 | ? |  |  |
| 10^1^ | 1.02 kb del | 1.02 kb del | ? | 7 | ? |  |  |
| 11^1^ | 1.02 kb del | 1.02 kb del | ? | 6 | ? |  |  |
| 12^2^ | 1.02 kb del | 1.02 kb del | ? | 6 | ? |  |  |
| 13^2^ | 1.02 kb del | c.998C>T | ? | 6 | ? |  |  |
| 14^3^ | 1.02 kb del | c.1135_1138delCTGT | ? | 5 | ? |  |  |
| 15^4^ | 1.02 kb del | c.374G>C | ? | 6 | ? |  |  |
| 16^4^ | 1.02 kb del | c.374G>C | ? | 6 | ? |  |  |
| 17^4^ | 1.02 kb del | 1.02 kb del | Yes | 5 | ? |  |  |
| 18^4^ | 1.02 kb del | 1.02 kb del | ? | 7 | ? |  |  |
| 19^4^ | c.622-623insT | c.622-623insT | Yes | ? | 2 |  |  |
| 20^4^ | 1.02 kb del | 1.02 kb del | ? | 5 | ? |  |  |
| 21^4^ | 1.02 kb del | 1.02 kb del | ? | ? | 7 |  |  |
| 22^4^ | c.265C>T | c.265C>T | ? | 8 | ? |  |  |
| 23^4^ | 1.02 kb del | 1.02 kb del | ? | ? | 6 |  |  |
| 24^4^ | c.370dupT | c.1001G>A | ? | 6 | ? |  |  |
| 25^5^ | 1.02 kb del | 1.02 kb del | ? | 7 | ? |  |  |
| 26^5^ | 1.02 kb del | 1.02 kb del | ? | 8 | 8 |  |  |
| 27^5^ | 1.02 kb del | 1.02 kb del | ? | 7 | 10 |  |  |
| 28^5^ | 1.02 kb del | 1.02 kb del | ? | 8 | 10 |  |  |
| 29^5^ | 1.02 kb del | 1.02 kb del | ? | 9 | 13 |  |  |
| 30^5^ | 1.02 kb del | 1.02 kb del | ? | 9 | 7 |  |  |
| 31^5^ | 1.02 kb del | 1.02 kb del | ? | 6 | 7 |  |  |
| 32^5^ | 1.02 kb del | 1.02 kb del | ? | 7 | ? |  |  |
| 33^5^ | 1.02 kb del | 1.02 kb del | ? | 7 | ? |  |  |
| 34^5^ | 1.02 kb del | 1.02 kb del | ? | 7 | ? |  |  |
| 35^6^ | 1.02 kb del | 1.02 kb del | ? | 5 | 5 |  |  |
| 36^6^ | 1.02 kb del | 1.02 kb del | ? | 5 | ? |  |  |
| 37^7^ | 1.02kb del | 1.02 kb del | ? |  |  | 6y: 87  7y: 88 | Not specified |
| 38^7^ | 1.02 kb del | 1.02 kb del | ? |  |  | 9y: 75  10y: 79 | Not specified |
| 39^7^ | 1.02 kb del | 1.02 kb del | ? |  |  | 17y: 43  18y: 43 | Not specified |
| 40^7^ | 1.02 kb del | 1.02 kb del | ? |  |  | 18y: 40  19y: 40 | Not specified |
| 41^7^ | 1.02 kb del | 2.8 kb del | ? |  |  | 7y: 80  8y: 88 | Not specified |
| 42^7^ | 1.02 kb del | 2.8 kb del | ? |  |  | 9y: 53  10y: 69 | Not specified |
| 43^8^ | 1.02 kb del | c.49G>T | ? | 5 | 5 |  |  |
| 44^9^ | 1.02 kb del | 1.02 kb del | Yes | 4 |  |  |  |
| 45^9^ | 1.02 kb del | 1.02 kb del | Yes | 7 |  |  |  |
| 46^10^ | 1.02 kb del | c.424delG | ? | ? | 3 |  |  |
| 47^11^ | 1.02 kb del | 1.02 kb del | ? |  |  | 6y: 85  11y: 79 | WISC-R |
| 48^11^ | 1.02 kb del | Not specified | ? |  |  | 9y: 80  14y: 74 | WISC-R |
| 49^11^ | 1.02 kb del | Not specified | ? |  |  | 7y: 84  12y: 72 | WISC-R |
| 50^11^ | 1.02kb del | 1.02 kb del | ? |  |  | 9y: 69  14y: 45 | WISC-R |
| 51^11^ | 1.02 kb del | 1.02 kb del | ? |  |  | 8y: 87  13y: 65 | WISC-R |
| 52^11^ | 1.02 kb del | 1.02 kb del | ? |  |  | 8y: 78  13y: 65 | WISC-R |
| 53^11^ | 1.02 kb del | 1.02 kb del | ? |  |  | 7y: 103  12y: 73 | WISC-R |
| 54^11^ | 1.02 kb del | Not specified | ? |  |  | 8y: 84  13y: 74 | WISC-R |
| 55^11^ | 1.02 kb del | Not specified | ? |  |  | 7y: 91  12y: 80 | WISC-R |
| 56^11^ | 1.02kb del | 1.02 kb del | ? |  |  | 6y: 105  11y: 75 | WISC-R |
| 57^11^ | 1.02 kb del | 1.02 kb del | ? |  |  | 6y: 91  11y: 87 | WISC-R |
| 58^11^ | 1.02 kb del | 1.02 kb del | ? |  |  | 7y: 94  12y: 77 | WISC-R |
| 59^11^ | 1.02 kb del | 1.02 kb del | ? |  |  | 10y: 49  15y: 45 | WISC-R |
| 60^12^ | 1.02 kb del | 1.02 kb del | ? | 4 | ? |  |  |
| 61^12^ | 1.02 kb del | 1.02 kb del | ? | 5 | ? |  |  |
| 62^12^ | 1.02 kb del | 1.02 kb del | ? | 4 | ? |  |  |
| 63^13^ | ? | ? | Yes | 6 | 6 |  |  |
| 64^14^ | 1.02 kb del | 2.8 kb deletion | ? | 7 | 8 | 8y: 105  12y: 80  16y: 70 | Not specified |
| 65^14^ | 1.02 kb del | 2.8 kb deletion | ? | 7 | ? | 6y: 84  12y: 67 | Not specified |
| 66^14^ | 1.02 kb del | 2.8 kb deletion | ? | 6 | ? |  |  |
| 67^14^ | 1.02 kb del | 2.8 kb deletion | ? | 5 | ? |  |  |
| 68^14^ | 1.02 kb del | 2.8 kb deletion | ? | 6 | ? |  |  |
| 69^14^ | 1.02 kb del | 2.8 kb deletion | ? | 5 | ? | 7y: 91  14y: 77 | Not specified |
| 70^14^ | 1.02 kb del | 2.8 kb deletion | ? | 5 | ? | 5y: 106 | Not specified |
| 71^14^ | 1.02 kb del | c.533+1G>C | ? | 7 | ? |  |  |
| 72^15^ | 1.02 kb del | 1.02 kb del | ? | 5 | ? |  |  |
| 73^16^ | 1.02 kb del | 1.02 kb del | ? | 7 | 8 |  |  |
| 74^17^ | 1.02 kb del | 1.02 kb del | ? | 5 | ? |  |  |
| 75^18^ | ? | ? | Yes | 8 | ? |  |  |
| 76^19^ | ? | ? | Yes | 6 | ? |  |  |
| 77^20^ | ? | ? | Yes | 7 | ? |  |  |
| 78^21^ | ? | ? | Yes | 9 | 9 |  |  |
| 79^22^ | ? | ? | Yes | 7 | ? |  |  |
| 80^23^ | ? | ? | Yes | 6 | 4 | 4y: 100  5y: 97  6y: 89 | WISC; WPPSI |
| 81^23^ | ? | ? | Yes | 7 | 5 | 5y: 92  6y: 101  7y: 79 | WISC; WPPSI |
| 82^24^ | ? | ? | Yes | 8 | ? |  |  |
| 83^24^ | ? | ? | Yes | 6 | ? |  |  |
| 84^25^ | ? | ? | Yes | 6 | ? |  |  |
| 85^26^ | ? | ? | Yes | 8 | ? |  |  |
| 86^27^ | ? | ? | Yes | 6 | 6 |  |  |
| 87^28^ | ? | ? | Yes | 8 | ? |  |  |
| 88^28^ | ? | ? | Yes | 7 | ? |  |  |
| 89^28^ | ? | ? | Yes | 9,5 | ? |  |  |
| 90^28^ | ? | ? | Yes | 7 | ? |  |  |
| 91^28^ | ? | ? | Yes | 4,5 | ? |  |  |
| 92^28^ | ? | ? | Yes | 7,5 | ? |  |  |
| 93^28^ | ? | ? | Yes | 5 | ? |  |  |
| 94^28^ | ? | ? | Yes | 6 | ? |  |  |
| 95^28^ | ? | ? | Yes | 5 | ? |  |  |
| 96^28^ | ? | ? | Yes | 7,5 | ? |  |  |
| 97^28^ | ? | ? | Yes | 5 | ? |  |  |
| 98^28^ | ? | ? | Yes | 6 | ? |  |  |
| 99^28^ | ? | ? | Yes | 5 | ? |  |  |
| 100^29^ | ? | ? | Yes | 5 | ? |  |  |
| 101^29^ | ? | ? | Yes | 6 | ? |  |  |
| 102^29^ | ? | ? | Yes | 6 | ? |  |  |
| 103^29^ | ? | ? | Yes | 7 | ? |  |  |
| 104^29^ | ? | ? | Yes | 9 | ? |  |  |

WISC = Wechsler Intelligence Scale for Children. Over time, different versions were used.
WPPSI = Wechsler Preschool and Primary Scale of Intelligence. **Supplementary table 2: Summary of patient characteristics systematic review – protracted CLN3**

| **Patient No** | **Mutation 1** | **Mutation 2** | **Vacuolated lymphocytes** | **Age onset visual decline**  **(years)** | **Age onset cognitive decline**  **(years)** | **IQ scores** | **IQ test method used** |
| --- | --- | --- | --- | --- | --- | --- | --- |
| 105^30^ | c.944-945dupA | c.1045_1050del | ? | 9 | 28 |  |  |
| 106^31^ | c.494G>A | c.494G>A | Yes | 7 | ? |  |  |
| 107^31^ | c.494G>A | c.494G>A | Yes | 7 | ? |  |  |
| 108^32^ | 1.02 kb del | c.883G>A | Yes | 6 | 35 |  |  |
| 109^7^ | 1.02 kb del | c.883G>A | ? | ? | ? | 11y: 103  12y: 119 | Not specified |
| 110^11^ | 1.02 kb del | Not specified | ? | ? | ? | 8y: 94  13y: 115 | WISC-R |
| 111^14^ | 1.02 kb del | c.1001G>A | Yes | 5 | ? |  |  |
| 112^33^ | ? | ? | Yes | ? | ? | 11y: 124  12y: 117  13y: 118  14y: 117  16y: 113  18y: 114 | Williams intelligence test for children with defective vision |
| 113^33^ | ? | ? | Yes | ? | ? | 10y: 97  11y: 97  12y: 94  13y: 97  15y: 92 | Williams intelligence test for children with defective vision |
| 114^34^ | 1.02 kb del | c.302T>C | ? | ? | ? |  |  |
| 115^34^ | 1.02 kb del | c.509T>C | Yes | 10 | ? |  |  |
| 116^35^ | 1.02 kb del | c.883G>A | ? | 5 | 45 |  |  |
| 117^35^ | 1.02 kb del | c.883G>A | Yes | 5 | ? |  |  |
| 118^36^ | ? | ? | Yes | 10 | 33 |  |  |

WISC = Wechsler Intelligence Scale for Children. Over time, different versions were used.
WPPSI = Wechsler Preschool and Primary Scale of Intelligence. **Supplementary table 3: Summary of patient characteristics referral center cohort – classical CLN3**

| **Patient No** | **Mutation 1** | **Mutation 2** | **Vacuolated lymphocytes** | **Age onset visual decline**  **(years)** | **Early school history** | **IQ scores** | **IQ test method used** |
| --- | --- | --- | --- | --- | --- | --- | --- |
| 119 | ? | ? | Yes | 5 | Aberrant <7y | 7y: test not completed | WISC-R |
| 120 | ? | ? | Yes | 6 | Aberrant <7y | 7y: 84 |  |
| 121 | 1.02 kb del | c.1000C>T | Yes | ? | Aberrant from 8y | X |  |
| 122 | 1.02 kb del | 1.02 kb del | Yes | 3 | ? | X |  |
| 123 | 1.02 kb del | c.1054C>T | Yes | 5 | Aberrant <7y | X |  |
| 124 | ? | ? | Yes | 6 | ? | X |  |
| 125 | 1.02 kb del | ? | Yes | 6 | Aberrant <7 y | X |  |
| 126 | 1.02 kb del | ? | Yes | 7 | Aberrant from 7-8y of age | X |  |
| 127 | 1.02 kb del | 1.02 kb del | Yes | 5 | Aberrant <7y | 8y: test not completed | WISC-R |
| 128 | 1.02 kb del | c.1A>C | Yes | 5 | Aberrant <7y | 6y: 71 | WPPSI-3 |
| 129 | 1.02 kb del | 1.02 kb del | Yes | 5 | Aberrant <7y | 8y: test not completed | WISC-R |
| 130 | c.1054C>T | c.1054C>T | Yes | 6 | Aberrant <7y | 7y: 69 | WISC-3 |
| 131 | Del exon 9-15 | Del exon 9-15 | Yes | 5 | Aberrant <7y | 7y: 57 | WISC |
| 132 | 1.02 kb del | 1.02 kb del | Yes | 4 | Aberrant <7y | 6y: 79 | WPPSI |
| 133 | 1.02 kb del | 1,02 kb del | Yes | 5 | Aberrant <7y | 7y: test not completed | WISC-3 |
| 134 | 1.02 kb del | 1.02 kb del | Yes | 4 | ? | X |  |
| 135 | 1.02 kb del | 1.02 kb del | Yes | ? | Aberrant <7y | 7y: 65 | WPPSI-R |
| 136 | 1.02 kb del | 1.02 kb del | Yes | 5 | Aberrant <7y | 6y: 71 | SON-R |
| 137 | 1.02 kb del | 1,02 kb del | Yes | 7 | Aberrant <7y | 7y: 71 | WISC-3 |
| 138 | 1.02 kb del | 1.02 kb del | Yes | 8 | Aberrant <7y | X |  |
| 139 | 1.02 kb del | 1.02 kb del | Yes | 5 | Aberrant <7y | 6y: 68 | WPPSI-3 |

WISC = Wechsler Intelligence Scale for Children. Over time, different versions were used.
WPPSI = Wechsler Preschool and Primary Scale of Intelligence.
SON = Snijders-Oomen non-verbal intelligence test.

**Supplementary references**

^1^ Dulz S, Wagenfeld L, Nickel M et al (2016) Novel morphological macular findings in juvenile CLN3 disease. Br J Ophthalmol 100(6):824-8.

^2^ Hansen MS, Hove MN, Jensen H, Larsen M (2016) OPTICAL COHERENCE TOMOGRAPHY IN JUVENILE NEURONAL CEROID LIPOFUSCINOSIS. Retin Cases Brief Rep 10(2):137-9.

^3^ Drack AV, Miller JN, Pearce DA (2013) A novel c.1135_1138delCTGT mutation in CLN3 leads to juvenile neuronal ceroid lipofuscinosis. J Child Neurol 28(9):1112-6.

^4^ Perez-Poyato MS, Mila Recansens M, Ferrer Abizanda I et al (2011) Juvenile neuronal ceroid lipofuscinosis: clinical course and genetic studies in Spanish patients. J Inherit Metab Dis 34:1083–1093.

^5^ Valadares-Freitas ER, Pizarro MX, Oliveira LR, et al (2011) Juvenile neuronal ceroid-lipofuscinosis: clinical and molecular investigation in a large family in Brazil. Arq Neuropsiquiatr 69:13-18.

^6^ Elkay M, Silver K, Penn RD, Dalvi A (2009) Dystonic storm due to Batten’s disease treated with pallidotomy and deep brain stimulation. Mov Disord 15;24(7):1048-53.

^7^ Aberg L, Talling M, Harkonen T et al (2008) Intermittent prednisolone and autoantibodies to GAD65 in juvenile neuronal ceroid lipofuscinosis. Neurology 70(14):1218-20.

^8^ Kwon JM, Rothberg PG, Leman AR, Weimer JM, Mink JW, Pearce DA (2005) Novel CLN3 mutation predicted to cause complete loss of protein function does not modify the classical JNCL phenotype. Neurosci Lett 387(2):111-4.

^9^ Mantel I, Brantley MA Jr, Bellmann C et al (2004) Juvenile neuronal ceroid lipofuscinosis (Batten disease) CLN3 mutation (Chrom 16p11.2) with different phenotypes in a sibling pair and low intensity in vivo autofluorescence. Klin Monbl Augenheilkd 221(5):427-30.

^10^ Vercammen L, Buyse GM, Proost JE, Van Hove JL (2003) Neuroleptic malignant syndrome in juvenile neuronal ceroid lipofuscinosis associated with low-dose risperidone therapy. J Inherit Metab Dis 26(6):611-2.

^11^ Lamminranta S, Aberg LE, Autti T et al (2001) Neuropsychological test battery in the follow-up of patients with juvenile neuronal ceroid lipofuscinosis. J Intellect Disabil Res 45(Pt 1):8-17.

^12^ Bohra LI, Weizer JS, Lee AG, Lewis RA (2000) Vision loss as the presenting sign in juvenile neuronal ceroid lipofuscinosis. J Neuroophthalmol 20(2):111-5.

^13^ Villanova M, Ceuterick C, Dotti MT et al (1999) Detection of beta-A4 amyloid and its precursor protein in the muscle of a patient with juvenile neuronal ceroid lipofuscinosis (Spielmeyer-Vogt-Sjogren). *Acta Neuropathol* 98(1):78-84.

^14^ Lauronen L, Munroe PB, Jarvela I et al (1999) Delayed classic and protracted phenotypes of compound heterozygous juvenile neuronal ceroid lipofuscinosis. *Neurology* 15;52(2):360-5.

^15^ Inan C, Wong D, Wisniewski KE, Rose AL, Musarella MA (1998) First African-American child with juvenile neuronal ceroid lipofuscinosis. *Am J Med Genet* 79(5):335-6.

^16^ Weleber RG (1998) The dystrophic retina in multisystem disorders: the electroretinogram in neuronal ceroid lipofuscinoses. *Eye (Lond)* 12(Pt 3b):580-90.

^17^ Aberg L, Jarvela I, Rapola J et al (1998) Atypical juvenile neuronal ceroid lipofuscinosis with granular osmiophilic deposit-like inclusions in the autonomic nerve cells of the gut wall. *Acta Neuropathol* 95(3):306-12.

^18^ Marshman WE, Lee JP, Jones B, Schalit G, Holder GE (1998) Duane's retraction syndrome and juvenile Batten's disease: a new association? *Aust N Z J Ophthalmol* 26(3):251-4.

^19^ Crow YJ, Tolmie JL, Howatson AG, Patrick WJ, Stephenson JB (1997). Batten disease in the west of Scotland 1974-1995 including five cases of the juvenile form with granular osmiophilic deposits. *Neuropediatrics* 28(3):140-4.

^20^ Lake BD, Steward CG, Oakhill A, Wilson J, Perham TG (1997). Bone marrow transplantation in late infantile Batten disease and juvenile Batten disease. *Neuropediatrics* 28(1):80-1.

^21^ Taschner PE, de Vos N, Thompson AD et al (1995) Chromosome 16 microdeletion in a patient with juvenile neuronal ceroid lipofuscinosis (Batten disease). *Am J Hum Genet* 56(3):663-8.

^22^ Horiguchi M, Miyake Y (1992) Batten disease--deteriorating course of ocular findings. *Jpn J Ophthalmol* 36(1):91-6.

^23^ Kristensen K, Lou HC (1983) Central nervous system dysfunction as early sign of neuronal ceroid lipofuscinosis. *Dev Med Child Neurol* 25(5):588-90.

^24^ Seeliger M, Rüther K, Apfelstedt-Sylla E, Schlote W, Wohlrab M, Zrenner E (1997) Juvenile neuronal ceroid lipofuscinosis (Batten-Mayou) disease. Ophthalmologic diagnosis and findings. *Ophthalmologe* 94(8):557-62.

^25^ Henry JG, Stevens SM (1982) Neuronal ceroid lipofuscinosis in the amaurotic retardate: electron microscopic confirmation. *Aust J Ophthalmol* 10(3):161-6.

^26^ Johnson J (1961) Juvenile amaurotic idiocy: a case report. *J Ment Sci* 107:931-5.

^27^ Piatella L, Cardinali C, Zamponi N, Papa O (1991) Spielmeyer-Vogt disease: clinical and neurophysiological aspects. *Child’s Nerv Syst* 7:226-230.

^28^ Jensen GE, Clausen J, Melchior JC, Konat G (1977) Clinical, social and biochemical studies on Batten's syndrome, alias Spielmeyer-Vogt or Stengel's syndrome. *Eur Neurol* 15(4):203-11.

^29^ Nardocci N, Verga ML, Binelli S, Zorzi G, Angelini L, Bugiani O (1995) Neuronal ceroid-lipofuscinosis: a clinical and morphological study of 19 patients. *Am J Med Genet* 5;57(2):137-41.

^30^ Licchetta L, Bisulli F, Fietz M t al (2015) A novel mutation of Cln3 associated with delayed-classic juvenile ceroid lipofuscinois and autophagic vacuolar myopathy. *Eur J Med Genet* 58(10):540-544.

^31^ Cortese A, Tucci A, Piccolo G et al (2014) Novel CLN3 mutation causing autophagic vacuolar mypopathy. *Neurology* 10;82(23):2072-6.

^32^ Aberg L, Lauronen L, Hämäläinen J, Mole SE, Autti T (2009) A 30-year follow-up of a neuronal ceroid lipofuscinosis patient with mutations in CLN3 and protracted disease course. *Pediatr Neurol* 40(2):134-7.

^33^ Bennett MJ, Gayton AR, Rittey CD, Hosking GP (1994) Juvenile neuronal ceroid-lipofuscinosis: developmental progress after supplementation with polyunsaturated fatty acids. *Dev Med Child Neurol* 36(7):630-8.

^34^ Munroe PB, Mitchison HM, O'Rawe AM et al (1997) Spectrum of mutations in the Batten disease gene, CLN3. *Am J Hum Genet* 61(2):310-6.

^35^ Wisniewski KE, Zhong N, Kaczmarski W et al (1998) Compound heterozygous genotype is associated with protracted juvenile neuronal ceroid lipofuscinosis. *Ann Neurol* 43(1):106-10.

^36^ Goebel HH, Pilz H, Gullota F (1976) The protracted form of juvenile neuronal ceroid-lipofuscinosis. *Acta Neuropathol* 36(4):393-6.
